# Clinical characteristics and prognosis of osteosarcoma in young children: a retrospective series of 15 cases

**DOI:** 10.1186/1471-2407-11-407

**Published:** 2011-09-24

**Authors:** Maud AM Guillon, Pierre MJ Mary, Laurence Brugière, Perrine Marec-Bérard, Hélène D Pacquement, Claudine Schmitt, Jean-Marc Guinebretière, Marie-Dominique P Tabone

**Affiliations:** 1 Department of Pediatric Oncology, Institut Curie, Paris, France; 2 Department of Pediatric Surgery, Hôpital Armand Trousseau, Paris, France; 3 Department of Pediatric Oncology, Institut Gustave Roussy, Villejuif, France; 4 Department of Pediatric Oncology, Centre Léon Bérard, Lyon, France; 5 Department of Pediatric Hematology and Oncology, Centre Hospitalo-Universitaire Brabois, Nancy, France; 6 Laboratory of Pathology, Centre René Huguenin, Saint-Cloud, France; 7 Department of Pediatric Hematology-Oncology, Hôpital Armand Trousseau, Paris, France

**Keywords:** osteosarcoma, young children, functional recovery, prognosis

## Abstract

**Background:**

Osteosarcoma is the most common primary bone malignancy in childhood and adolescence. However, it is very rare in children under 5 years of age. Although studies in young children are limited in number, they all underline the high rate of amputation in this population, with conflicting results being recently reported regarding their prognosis.

**Methods:**

To enhance knowledge on the clinical characteristics and prognosis of osteosarcoma in young children, we reviewed the medical records and histology of all children diagnosed with osteosarcoma before the age of five years and treated in SFCE (Société Française des Cancers et leucémies de l'Enfant) centers between 1980 and 2007.

**Results:**

Fifteen patients from 7 centers were studied. Long bones were involved in 14 cases. Metastases were present at diagnosis in 40% of cases. The histologic type was osteoblastic in 74% of cases. Two patients had a relevant history. One child developed a second malignancy 13 years after osteosarcoma diagnosis.

Thirteen children received preoperative chemotherapy including high-dose methotrexate, but only 36% had a good histologic response. Chemotherapy was well tolerated, apart from a case of severe late convulsive encephalopathy in a one-year-old infant. Limb salvage surgery was performed in six cases, with frequent mechanical and infectious complications and variable functional outcomes.

Complete remission was obtained in 12 children, six of whom relapsed. With a median follow-up of 5 years, six patients were alive in remission, seven died of their disease (45%), in a broad range of 2 months to 8 years after diagnosis, two were lost to follow-up.

**Conclusions:**

Osteosarcoma seems to be more aggressive in children under five years of age, and surgical management remains a challange.

## Background

Osteosarcoma mainly occurs in teenagers and young adults. It is the most frequent primary bone malignancy in children and adolescents under 24 years of age, with an annual incidence of 4.4 cases per million in the United States [1]. In France, the annual incidence is 3.6 cases per million children under 15 years of age [2] and 9.2 per million adolescents aged from 15 to 19 years [3]. These patients' prognosis has improved markedly over the last three decades, with the use of multiagent chemotherapy and the frequency of amputation decreased over time with the advance of endoprosthesis technology and conservative surgery. Several studies have attempted to assess the impact of age on prognosis, comparing survival rates in children under the ages of 10, 12 or 14 years with those obtained in older patients with current therapies [4-6]. In 2003, Nagarajan et al reported comparable outcomes for children who presented with localised osteosarcoma at the age of 10 years or less and for those diagnosed at older age [4]. However, until recently, only limited series and case reports have been described in very young children [7-13]. Last year, several groups reported studies focused specifically on children under five years old. They represent only 1 to 2.8% of cases, but the rate of amputation remains high in these children [14-16]. Regarding prognosis, conflicting results were reported: some authors finding inferior outcomes compared to older children, even using a Cox proportional hazards model controlling for metastatic status [15], others finding that survival was in the range of that observed for older patients [14,16]. However, in the Italian series, patients with metastases at diagnosis were excluded.

The aim of this retrospective study was to enhance knowledge on the clinical characteristics of osteosarcoma in children under 5 years of age and to elucidate whether there is any difference with regards to the outcome between very young and older patients. We specifically investigated whether children who developed osteosarcoma so early in their life had underlining condition and could have a different tumour biology, how many had disseminated disease at diagnosis and how they responded to and tolerated chemotherapy. The functional outcome of lower-limb surgery was also examined as surgical management of these children remains a challenge.

## Methods

This study was approved by the DIUOP (pediatric oncology inter university graduation, coordinated by G. Vassal, MD, PhD) educational committee (including scientific and ethic evaluation of all research projects). We analysed the medical records of all children under five years of age at diagnosis of osteosarcoma who were treated in member centers of the Société Française de lutte contre les Cancers et leucémies de l'Enfant et de l'adolescent (SFCE) between 1980 and 2007.

The following information was collected: clinical characteristics (age at diagnosis, sex, medical background and genetic predisposition to cancer); tumour characteristics (location, size, metastatic status at diagnosis); treatment (preoperative chemotherapy, surgery, post-operative treatment, adverse effects); and outcome (remission, relapse, survival). Functional status was obtained from the patients' orthopaedists. The histologic diagnoses were confirmed by centralized slide review. Survival was calculated using the Kaplan-Meir method, with standard errors [17]. Overall survival was calculated from diagnosis until death and event free survival until progression for patients who never achieved complete remission, or relapse for those who had complete surgical resection of the tumour.

## Results

Fifteen children (7 girls and 8 boys) under 5 years of age were treated for osteosarcoma between January 1980 and December 2007 in 7 SFCE centers. They were aged between 1.0 and 4.9 years (median 3.9 years).

Two patients had relevant histories:

- A 3-year-old girl had constitutional tall height (105.5 cm at diagnosis, +4 SD), with no etiologic diagnosis

- A 4.2-year-old boy had a polymalformative syndrome with intrauterine growth retardation, microcephaly and blindness. He had received growth hormone for three years before osteosarcoma onset.

Only one patient, a one-year-old boy, had molecular studies, which showed normal Rb and P53 gene status. None of the patients had a family history of cancer.

Clinical features are described in table 1. Pain was the most frequent presenting symptom (n = 12). Swelling was prominent in two cases, and one pathological fracture occurred after a mistaken treatment for a bone cyst. The median duration of symptoms before diagnosis was 53 days (15-180 days) overall, and respectively 56 and 60 days in patients with metastatic and localized forms. The primary tumour was located on a long bone in 14 cases (femur, n = 11, 73%; tibia, n = 1; humerus, n = 2). The tumour involved the whole bone in 2 cases. The diaphysis was involved alone in 4 cases, and the epiphysis in 8 cases. One tumour affected a rib. In the 14 patients with long-bone primary tumours, the median dimensions were 54 mm (28-106 mm) by 36.4 mm (10-50 mm). Six patients (40%) had pulmonary metastasis at diagnosis. None of the patients had metastasis of bone or other tissues at diagnosis.

**Table 1:** Patients characteristics at diagnosis

Patient	Year range of diagnosis	Age range at diagnosis (years)	Duration range of symptoms (months)	Metastases at diagnosis
1		2-3	NA	yes
2		3-4	<2	yes
3	1980 -1989	3-4	<2	no
4		3-4	<2	no
5		4-5	>4	no
6		4-5	2-4	yes

7		4-5	<2	no
8	1990 -1999	4-5	2-4	no
9		3-4	<2	no

10		4-5	<2	yes
11		3-4	2-4	no
12	2000 -2007	4-5	<2	yes
13		1-2	<2	no
14		4-5	<2	no
15		2-3	2-4	yes

NA: data non-available

Centralized slide review showed that 12 tumours were conventional osteoblastic osteosarcomas, while one was mixed (osteoblastic, chondroblastic and fibroblastic), one was telangiectasic, and one was fibroblastic.

Treatments are summarized in table 2. Thirteen children received preoperative chemotherapy. Nine patients received previously described regimens, consisting of the Rosen T10 protocol in 1 case [18], HELP in 2 cases [19], Os 87 in 2 cases [20], and Os 94 in 4 cases [21]. Two patients received the French Os 2005 regimen preoperatively, consisting of seven courses of high-dose methotrexate (HDMTX: 12 g/m^2^) with leucovorin rescue and two courses of etoposide (300 mg/m^2^) and ifosfamide (12 g/m^2^). Patients with good response to chemotherapy received 12 courses of HDMTX and 3 courses of etoposide and ifosfamide post-operatively, while patients with poor response to chemotherapy received 10 courses of HDMTX and 5 courses of cisplatin (CDDP, 120 mg/m^2^) plus doxorubicin (75 mg/m^2^). One patient received HDMTX and vincristine before surgery, and one patient received HDMTX and doxorubicine. Two patients received no preoperative treatment.

**Table 2:** treatment and evolution of patients

Patient	Preoperative chemotherapy	Surgery	% Viable tumor cells	Post operative chemotherapy	Relapse	Status
1	no	desarticulation of the hip	NAp	no	NAp	dod

2	HD MTX, VCR	amputation	NA	CDDP	NAp	dod

3	T10	desarticulation	100%	DOX, CDDP, bleo, actinomycin, cyclo	lung	dod

4	os 87	resection-reconstruction	30%	Ifosfamide, vindesine	local	Lost to follow-up

5	HD MTX, DOX	resection-reconstruction	10%	DOX, HD MTX	no	CR1

6	HELP	tigh's amputation	3%	HELP	bone	dod

7	os 87	amputation	60%	os87	lung	dod

8	HELP	Trans-iliac amputation	8%	HELP	no	CR1 second cancer

9	os 94	resection-prothesis	80%	VP16, ifosfamide	lung	dod

10	os 94	amputation	>50%	os 94	local	dod

11	os 94	resection-reconstruction	15%	os 94	no	CR1

12	os 94	resection-prothesis	0%	os 94	no	CR1

13	no	costectomy	NAp	os 94	no	CR1

14	os 05	resection-prothesis	8%	os 05	no	CR1

15	os 05	NA	NA	NA	NA	Lost to follow-up

dod: Dead of disease, CR1: first complete remission, NA: data non available, Nap: data non applicable, VCR: vincristine, DOX: doxorubicine, HDMTX: High dose Methotrexate, CDDP: cisplatin, bleo: bleomycin, cyclo: cyclophosphamide.

The tumour progressed in 5 cases during pre-operative treatment, which was halted. Surgery was conservative in 6 (40%) of these 13 children who received preoperative chemotherapy, while six patients were amputated and one was lost to follow-up when the tumour progressed. Conservative surgery consisted in resection and reconstruction by: prosthesis in 2 cases, expanding endoprosthesis in one case, vascularised fibula in 2 cases, and other type of autologous bone graft in 1 case. A total number of 14 patients underwent local surgery, 13 of them had microscopic complete tumour resection and for the patient remaining, quality of resection was not available. The histologic response was available in 11 cases. Only 4 patients (36%) had a good histologic response to chemotherapy, with less than 10% of viable tumour cells. Two of these patients had Huvos grade III or IV responses [18].

Post-operative chemotherapy is summarized in table 2. Chemotherapy was complicated by two cases of septicaemia, one electrolyte disorder, and one case of grade III hepatic toxicity.

**Figure 1:**
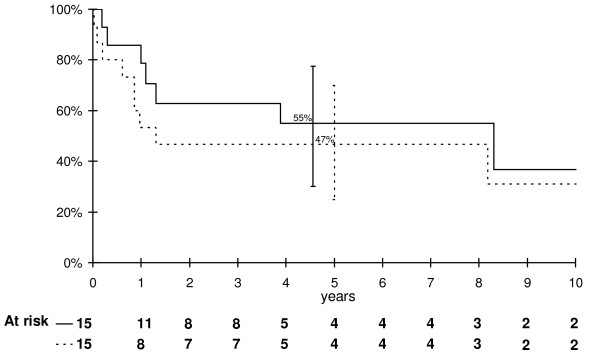
**Overall (———) and event-free (- - -) survival of the whole group of patients**. Vertical bars denote 95% confidence intervals at 5 years.

Overall and event free survival curves of the whole group of patients are shown in Figure 1. The overall survival rate at 5 years was 55% (95% confidence interval 30-78%), the event-free survival was 47% (95% confidence interval 23-70%). Out of 15 patients, 12 achieved a complete remission, but 6 of them had recurrences, in lung (n = 3) or bone (n = 3). One child was successfully treated for renal adenocarcinoma, which occurred 14 years after osteosarcoma diagnosis. With a median follow-up of 5 years, six patients were alive in first complete remission with no evidence of disease. Two patients were lost to follow-up with progressive disease, and 7 died of their disease. The median time between diagnosis and death was 12 months (range 2 months to 8 years).

We also examined functional outcome in the 9 children who survived for more than one year after diagnosis. One child had ablation of a rib, with no mechanical or pulmonary complications 3 years after diagnosis. One patient had a good functional result after tumour resection of the upper third of the humerus, reconstructed with a cement spacer. Three children had lower-limb-sparing surgery with reconstruction by vascularised fibula (2 cases) and growth's prosthesis (1 case). They were re-operated on between one and five times, for infections, prosthesis fracture, pseudoarthrosis, unequal limb length or varisation. One patient had a good functional result eight years after the first operation, the second patient still needed an orthesis for walking 5 years later, and the third patient had very poor knee-joint function and a length discrepancy of 5.5 cm. Among the four amputated children, two had transiliac or inter-ilio-abdominal amputation with acceptable functional results after prosthetic rehabilitation 6 months to 1 year after surgery. One boy had thigh amputation and was unable to receive prosthetic rehabilitation, owing to mental retardation. The other patient with thigh amputation was able to walk unassisted with prosthesis.

Chemotherapy was generally well tolerated: cardiac and auditory function remained normal in all the children who received doxorubicin or CDDP. Nevertheless, the youngest child, a one-year-old infant, suffered severe neurologic toxicity. He first developed severe peripheral neuropathy (despite not receiving vincristine or cisplatin) five months after ifosfamide administration. He then developed severe late convulsive encephalopathy two years after the end of chemotherapy, with white substance signal abnormalities on MRI, possibly attributable to HDMTX.

## Discussion

Osteosarcoma is exceedingly rare in young children. In France, Desandes et al found an incidence of 9.2 cases per million adolescents [3], compared to only 0.4 per million under-fives [2]. In our small series of 15 patients the sex ratio was 1.1 in favor of males, compared to 2.1 in adolescents in France [3]. The tumour arose in the diaphysis in 6 cases, compared to only 4% of cases in Pakos' series of 2680 patients [22].

Factors predisposing to cancer are a question of interest in patient developing an osteosarcoma at young age. No genetic predisposition was found in our population, but germinal mutation of Rb and p53 have only been investigated in one child. Another 3-year-old child had constitutional tall height (+4 SD). Osteosarcoma usually occurs during periods of rapid growth, but the relationship with height remains controversial, recent retrospective studies having given contradictory results. Buckley found no consistent relation with height in a case-control study of 152 children with osteosarcoma [23], while Gelberg found a significant positive association with height one year before diagnosis [24]. Adult patients with acromegaly have been reported to be subject to osteosarcoma [25]. In our series, one child was treated with growth hormone (GH) for 3 years before diagnosis, following intrauterine growth retardation and a polymalformative syndrome, without biological GH deficiency. Due to this syndrome, a genetic predisposition to tumour cannot be excluded. However isolated cases of osteosarcoma have been reported during GH treatment, but no formal relationship has been established [26,27]. Carel et al reported that GH-treated children had a relative risk of 13.8 for bone cancer [28]. The benefit of GH treatment probably outweighs the risks, but the indication should be examined closely in case of personal or familial risk factors.

The average interval between symptom onset and diagnosis was 2 months in our patients, in keeping with other studies [13,29], but longer than in Kager et al report [16]. Two children had mistakenly been treated for benign tumours, by infiltration or resection. Although benign tumours are more frequent than neoplasms in young children [30], the possibility of malignancy must be kept in mind.

Metastasis was found at diagnosis in 40% of the children in this study, a rate higher than reported in older children (10 to 20%) [31], or in young children by other groups [15,16].

The osteoblastic histologic type predominated in our series (73%), as in older patients and in the recent german study in young children [16]. Other studies found fibroblastic type [14] or telangiectasic type [15] as predominant subtype. However, in those studies, centralized slide review was not performed.

As this study spans a lengthy period, treatment was heterogeneous. However, all the children who received chemotherapy (14 out of 15) were treated with drugs known to be effective in osteosarcoma (HDMTX, ifosfamide, cisplatin or doxorubicin). A good tumour response to pre-operative chemotherapy was obtained in only 36% of our patients, while similar regimens have been reported to give good responses in 56% to 64% of patients with non-metastatic osteosarcoma [19,21,32] and in 42% of patients with metastatic forms [29]. This would suggest that preoperative chemotherapy is less effective in younger children. Likewise, Cho [33] reported good responses in only 2 (20%) of 10 children under 7 years of age. These results, together with the high frequency of metastases at diagnosis promote the hypothesis of a different tumour biology in young children, with more aggressive disease. An other explanation could be differences in chemotherapy metabolism in young patients. It has been demonstrated that systemic methotrexare and doxorubicin clearance tended to be lower in very young children [34], and quite recently, Crews et al found that in children and young adults with osteosarcoma, a lower methotrexate clearance was associated with lower probability of survival [35]. However recent studies did not find any difference in histological response in young patients compared to older patients [14,16] and previous methotrexate pharmacokinetics analyses stated that high peak level were associated with better outcome [36].

No major acute adverse effects of chemotherapy were noted, but a one-year-old boy developed late neurotoxicity. His peripheral neuropathy was attributable to ifosfamide, which is known to provoke painful peripheral sensory neuropathy [37], while his late convulsive encephalopathy and mental regression were attributable to HDMTX. Severe leukoencephalopathy has been described with this drug and cannot be avoided by folinic acid supplementation [37].

As reported by others in young children, we found a high amputation rate in our study, explained by tumour progression in some cases (n = 5), but also by the complexity of surgical reconstruction in young patients, and by the lengthy study period. Indeed, the frequency of amputation appears to be decreasing over time: it was 51% in a French study in 1988 [38] and only 6% in the Os 94 trial [21].

Owing to the small number of patients and the lack of quality-of-life assessment, the functional outcome of children who had lower-limb surgery was difficult to assess. The 3 children who had limb-sparing surgery had a high rate of mechanical complications. Skeletal maturity is an important determinant of functional outcome in children with greater growth potential. Limb-sparing procedures are more problematic than in adults, and remain a challenge in very young children [13-16,32,39].

The overall and event-free survival rates in this study are difficult to interpret, given the small number of patients, but our results seem similar to those reported by Kager et al [16]. It is noteworthy that 7 children (45%) died of their disease. Currently, the reported survival rate among children treated for osteosarcoma is about 19% for metastatic patients [29] and 76% at 5 years for non metastatic patients [21]. In Germany, Bielack et al reported an overall survival rate of 65% at 5 years [32]. In the US study by Mirabello et al, the overall survival rate was 61% at 5 years between 1973 and 2004 among patients under 24 years of age [1]. In very young children, some authors did not find difference in survival compared to older patients [12-14,16]. However, in a registries based study, Worch et al reported that in non metastatic patients, 5-year overall survival estimate was 51.9% for children who were 5 years of age or younger at diagnosis versus 67.3% for patients ages 6-19 years [15]. Metastasis at diagnosis is a well-known factor of poor prognosis. In our series only 3 out of 6 metastatic patients were alive at the cut-off date for this analysis. The response to neoadjuvant chemotherapy was also found to have a prognostic influence in most studies. In our study, 1 of the 4 patients who had a good response died, compared to 4 of the 7 patients with a poor response. Kager et al also found a negative impact of poor histological response on survival [16]. Six (50%) of the 12 children who obtained full remission in our series relapsed. This is a higher rate than in the Hartford's series of young children treated in the United States (25%) [13], but the same observation was done in Kager's study, who reported 11 recurrences among 23 patients (48%) who achieved complete remission [16]. Our results confirm that pattern of recurrence seems to be similar to older children with lung and bone involvement.

## Conclusion

Despite the small number of patients in this series, our findings provide further information on the characteristics of osteosarcoma in children under 5 years of age. In particular, this tumour frequently arises in the diaphysis of long bones, and seems to be more aggressive than in older patients, with a high frequency of metastasis at diagnosis and a poor response to chemotherapy, leading to poor survival. Given the growth potential in this age range, it is crucial to carefully consider local treatment options and their likely functional outcome.

## Competing interests

The authors declare that they have no competing interests.

## Authors' contributions

MG participated in study design, data acquisition and analysis, and drafted the manuscript, PM participated in quality control, data analysis and interpretation, LB participated in data acquisition, interpretation and statistical analysis, PMB participated in data acquisition and interpretation, HP participated in data acquisition and interpretation, CS participated in data acquisition, JMG carried out the histologic slides review, MDT conceived of the study, and participated in its design, coordination, and data analysis. All authors read and approved the final manuscript

## Pre-publication history

The pre-publication history for this paper can be accessed here:

http://www.biomedcentral.com/1471-2407/11/407/prepub

## Aknowledgement

We thanks

- the pediatric oncologists who provided clinical data for their center cases: Philippe Le Moine, University Hospital, Brest, France; Cecile Habay, University Hospital, Rennes, France;

- the pathologists who provided the histologic slides for centralized review: Patrick Josset, Armand Trousseau Hospital, Paris, France, Philippe Terrier, Gustave Roussy Institute, Villejuif, France;

- the orthopaedists who provided data about the functional outcome of children: Karima Abelin, Necker hospital, Paris, France; Laurent Barba, Clinique du Parc, Lyon, France; Madeleine Chapuis, University Hospital, Rennes, France; Christophe Glorion, Necker Hospital, Paris, France; Pierre Lascombe, University Hospital, Nancy, France; Eric Mascard, Saint Vincent de Paul Hospital, Paris, France; Jerôme Sales de Gauzy, University Hospital, Toulouse, France.

## References

[B1] Mirabello L Troisi RJ Savage SA Osteosarcoma incidence and survival rates from 1973 to 2004 Cancer 2009 115 1531 1543 10.1002/cncr.24121 19197972 PMC2813207

[B2] Desandes E Clavel J Berger C Bernard JL Blouin P de Lumley L Demeocq F Freycon F Gembara P Goubin A Le Gall E Pillon P Sommelet D Tron I Lacour B Cancer incidence among children in France, 1990-1999 Pediatr Blood Cancer 2004 43 749 757 10.1002/pbc.20148 15390289

[B3] Desandes E Lacour B Sommelet D Buemi A Danzon A Delafosse P Grosclaude P Mace-Lesech J Raverdy-Bourdon N Tretarre B Velten M Brugieres L Cancer incidence among adolescents in France Pediatr Blood Cancer 2004 43 742 748 10.1002/pbc.20106 15390305

[B4] Nagarajan R Weigel BJ Thompson RC Perentesis JP Osteosarcoma in the first decade of life Med Pediatr Oncol 2003 41 480 483 10.1002/mpo.10403 14515397

[B5] Meyers PA Heller G Healey J Huvos A Lane J Marcove R Applewhite A Vlamis V Rosen G Chemotherapy for nonmetastatic osteogenic sarcoma: the Memorial Sloan-Kettering experience J Clin Oncol 1992 10 5 15 1370176 10.1200/JCO.1992.10.1.5

[B6] Bacci G Ferrari S Mercuri M Longhi A Capanna R Tienghi A Brach del Prever A Comandone A Cesari M Bernini G Picci P Neoadjuvant chemotherapy for extremity osteosarcoma--preliminary results of the Rizzoli's 4th study Acta Oncol 1998 37 41 48 10.1080/028418698423168 9572653

[B7] Siegal GP Dahlin DC Sim FH Osteoblastic osteogenic sarcoma in a 34 month-old girl Am J Clin Pathol 1975 63 886 890 1056700 10.1093/ajcp/63.6.886

[B8] Levy ML Jaffe N Osteosarcoma in early childhood Pediatrics 1982 70 302 303 6954451

[B9] Luiz CP D'Orth BS Al Kharusi W Sethu AU Buhl L Al Lamki Z Osteosarcoma in a 26-month-old girl Cancer 1992 15 894 896 10.1002/1097-0142(19920815)70:4<894::aid-cncr2820700428>3.0.co;2-e 1643623

[B10] Sanchi-Alfonso V Fernandez-Fernandez CI Donat J Lombart-Bosch A Osteoblastic osteosarcoma in a 13-month-old girl Pathol Res Pract 1994 190 207 210 8058575 10.1016/S0344-0338(11)80713-2

[B11] Rivera-Luna R De Leon-Bojorge B Ruano-Aguilar J Castellanos A Vazquez C Osteosarcoma in children under three years of age Med Pediatr Oncol 2004 41 99 100 10.1002/mpo.10281 12764766

[B12] Kozakewich H Perez-Atayde AR Goorin AM Wilkinson RH Gebhardt MC Vawter GF Osteosarcoma in young children Cancer 1991 67 638 642 10.1002/1097-0142(19910201)67:3<638::AID-CNCR2820670319>3.0.CO;2-T 1985758

[B13] Hartford CM Wodowski KS Rao BN Khoury JD Neel MD Daw NC Osteosarcoma among children aged 5 years or younger J Pediatr Hematol Oncol 2006 28 43 47 16394893

[B14] Abate ME Longhi A Galetti S Ferrari S Bacci G Non-metastatic osteosarcoma of the extremities in children aged 5 years or younger Pediatr Blood Cancer 2010 55 652 654 10.1002/pbc.22567 20806363

[B15] Worch J Matthay K Neuhaus J Goldsby R Dubois SG Osteosarcoma in children 5 years of age or younger at initial diagnosis Pediatr Blood Cancer 2010 55 285 289 10.1002/pbc.22509 20582978 PMC2917386

[B16] Kager L Zoubek A Dominkus M Lang S Bodmer N Jundt G Klingebiel T Jürgens H Gadner H Bielack S COSS Study Group Osteosarcoma in very young children: experience of the Cooperative Osteosarcoma Study Group Cancer 2010 116 5316 5324 10.1002/cncr.25287 20672353

[B17] Kaplan EL Meier P Non parametric estimation from incomplete observations J Am Stat Assoc 1958 53 457 481 10.2307/2281868

[B18] Rosen G Caparros B Huvos AG Kosloff C Nirenberg A Cacavio A Marcove RC Lane JM Metha B Urban C Preoperative chemotherapy for osteogenic sarcoma: selection of postoperative adjuvant chemotherapy based on the response of the primary tumor to preoperative chemotherapy Cancer 1982 15 1221 1230 10.1002/1097-0142(19820315)49:6<1221::aid-cncr2820490625>3.0.co;2-e 6174200

[B19] Philip T Iliescu C Demaille MC Pacquement H Gentet JC Krakowski I Soler-Michel P Thiesse P Chauvin F Blay JY Brunat-Mentigny M High dose methotrexate and HELP-doxorubicine in non-metastatic osteosarcoma of the extremity: a french multicentre pilot study Ann Oncol 1999 10 1065 1071 10.1023/A:1008395126800 10572604

[B20] Tabone MD Kalifa C Rodary C Raquin M Valteau-Couannet D Lemerle J Osteosarcoma recurrences in pediatric patients previously treated with intensive chemotherapy J Clin Oncol 1994 12 2614 2620 7989936 10.1200/JCO.1994.12.12.2614

[B21] Le Deley MC Guinebretière JM Gentet JC Pacquement H Pichon F Marec-Bérard P Entz-Werlé N Schmitt C Brugière L Vanel D Dupoüy N Tabone MD Kalifa C Société Française d'Oncologie Pédiatrique (SFOP) SFOP OS94: a randomised trial comparing preoperative high-dose methotrexate plus doxorubicine to high dose methotrexate plus etoposide and ifosfamide in osteosarcoma patients Eur J Cancer 2007 43 752 761 10.1016/j.ejca.2006.10.023 17267204

[B22] Pakos EE Nearchou AD Grimer RJ Koumoullis HD Abudu A Bramer JA Jeys LM Franchi A Scoccianti G Campanacci D Capanna R Aparicio J Tabone MD Holzer G Abdolvahab F Funovics P Dominkus M Ilhan I Berrak SG Patino-Garcia A Sierrasesumaga L San-Julian M Garraus M Petrilli AS Filho RJ Macedo CR Alves MT Seiwerth S Nagarajan R Cripe TP Ioannidis JP Prognostic factors and outcomes for osteosarcoma: an international collaboration Eur J Cancer 2009 45 2367 2375 10.1016/j.ejca.2009.03.005 19349163

[B23] Buckley JD Pendergrass TW Buckley CM Pritchard DJ Nesbit ME Provisor AJ Robison LL Epidemiology of osteosarcoma and Ewing sarcoma in childhood Cancer 1998 83 1440 1448 10.1002/(SICI)1097-0142(19981001)83:7<1440::AID-CNCR23>3.0.CO;2-3 9762947

[B24] Gelberg KH Fitzgerald EF Hwang S Dubrow R Growth and development and other risk factors for osteosarcoma in children and young adults Int J Epidemiol 1997 26 272 278 10.1093/ije/26.2.272 9169161

[B25] Lima GA Gomes EM Nunes RC Vieira Neto L Sieiro AP Brabo EP Gadelha MR Osteosarcoma and acromegaly: a case report and review of the literature J Endocrinol Invest 2006 29 1006 1011 17259799 10.1007/BF03349215

[B26] Darendeliler F Bundak R Eriyilmaz SK Günöz H Baş F Saka N Follow-up eight year after discontinuation of growth hormone treatment in children with intrauterine growth retardation J Pediatr Endocrinol Metab 2002 15 795 800 10.1515/JPEM.2002.15.6.795 12099389

[B27] Buchanan CR Preece M Milner RDG Mortality, neoplasia and Creutzfeld-Jakob disease in patients treated with human pituitary hormone in the U K British Medical J 1991 302 824 828 10.1136/bmj.302.6780.824 PMC1669149 2025705

[B28] Carel JC Coste J Long-term safety of recombinant growth hormone Arch Ped 2007 14 615 617 10.1016/j.arcped.2007.04.004 17459678

[B29] Mialou V Philip T Kalifa C Perol D Gentet JC Marec-Berard P Pacquement H Chastagner P Defaschelles AS Hartmann O Metastatic osteoarcoma at diagnosis Cancer 2005 104 1100 1109 10.1002/cncr.21263 16015627

[B30] Senac MO Isaacs H Gwinn JL Primary lesion of bone in the first decade of life: rerospective survey of biopsy results Radiology 1986 160 491 495 3487812 10.1148/radiology.160.2.3487812

[B31] Kaste SC Pratt CB Cain AM Jones-Wallace DJ Rao BN Metastases detected at the time of diagnosis of primary pediatric extremity osteosarcoma at diagnosis: imaging features Cancer 1999 86 1602 1608 10.1002/(SICI)1097-0142(19991015)86:8<1602::AID-CNCR31>3.0.CO;2-R 10526292

[B32] Bielack S Kempf-Bielack B Delling G Exner GU Flege S Helmke K Kotz R Salzer-Kuntschik M Werner M Winkelmann W Zoubek A Jürgens H Winkler K Prognostic factors in high-grade osteosarcoma of the extremities or trunk J Clin Oncol 2002 20 776 790 10.1200/JCO.20.3.776 11821461

[B33] Cho WH Lee SY Son WS Park JH Osteosarcoma in pre-adolescent patients J Int Med Res 2006 34 676 681 17295001 10.1177/147323000603400614

[B34] McLeod HL Relling MV Crom WR Silverstein K Groom S Rodman JH Rivera GK Crist WM Evans WE Disposition of antineoplastic agents in the very young child Br J Cancer 1992 66 Suppl 18 S23 29 PMC2149660 1503923

[B35] Crews KR Liu T Rodriguez-Galindo C Tan M Meyer WH Panetta JC Link MP Daw NC High-dose methotrexate pharmacokinetics and outcome of children and young adults with osteosarcoma Cancer 2004 100 1724 1733 10.1002/cncr.20152 15073863

[B36] Graf N Winkler K Betlemovic M Fuchs N Bode U Methotrexate pharmacokinetics and prognosis in osteosarcoma J Clin Oncol 1994 12 1443 1451 8021736 10.1200/JCO.1994.12.7.1443

[B37] Verstappen CP Heimans JJ Hoekman K Postma TJ Neurotoxic complications of chemotherapy in patients with cancer Drugs 2003 63 1549 1563 10.2165/00003495-200363150-00003 12887262

[B38] Kalifa C Mlika N Dubousset J Contesso G Vanel D lumbroso J Experience with the T10 protocol in the pediatric departement of the Gustave Roussy Institute Bull Cancer 1988 75 207 211 2451956

[B39] Renard AJ Veth RP Schreuder HW van Loon CJ Koops HS van Horn JR Function and complications after ablative and limb-salvage therapy in lower extremity sarcoma of bone J Surg Oncol 2000 73 198 205 10.1002/(SICI)1096-9098(200004)73:4<198::AID-JSO3>3.0.CO;2-X 10797332

